# P082: Beijing and non-Beijing genotypes detection of mycobacterium tuberculosis by melting curve analyzes

**DOI:** 10.1186/2047-2994-2-S1-P82

**Published:** 2013-06-20

**Authors:** Z Tavakoli, A Nazemi

**Affiliations:** 1 Microbiology, Tonekabon Azad University, Tonekabon, Iran, Islamic Republic Of

## Introduction

*Tuberculosis* is one of the most important infectious diseases in the world today. Rapid diagnosis of drug resistant *Mycobacterium tuberculosis* (MTB) is critical to starting of an appropriate treatment and preventing of more spread drug resistant MTB strains.

## Objectives

Due to association of Beijing genotype with drug resistance in MTB, we developed a rapid and non-culture method for detection Beijing and non-Beijing MTB in clinical samples.

## Methods

We modified Taqman Real time PCR for detection Beijing and non-Beijing genotypes of Mycobacterium tuberculosis to a free probe method in presence of a single dye together with a melting curve analysis. We then performed a blinded screening with both methods on 33 septum samples from treated tuberculosis patients.

## Results

We were obtained the same results by both methods. Of the 33 patients, 5 samples were Beijing genotype and 28 were non-Beijing genotype. In free probe method, we were clearly identified a melting peak at 81°C corresponds to non-Beijing and a melting peak at 88°C corresponds to Beijing genotype.

## Conclusion

DNA melting curve analysis is a simple and efficient method for the specific detection of amplified products and greatly reduces the cost molecular detection.

## Disclosure of interest

None declared.

